# Emerging Epigenetic Therapeutics and Diagnostics for Autism Spectrum Disorder

**DOI:** 10.3390/cimb47070491

**Published:** 2025-06-27

**Authors:** Cassie Kao, Kim Kiat Lim, Ee Sin Chen

**Affiliations:** 1 Department of Biochemistry, Yong Loo Lin School of Medicine, National University of Singapore, Singapore 117596, Singapore; cassie.kao@duke.edu (C.K.); bchlimk@nus.edu.sg (K.K.L.); 2 National University Health System (NUHS), Singapore 119228, Singapore; 3 NUS Center for Cancer Research, Yong Loo Lin School of Medicine, National University of Singapore, Singapore 117599, Singapore; 4 NUS Artificial Intelligence Institute (NAII), National University of Singapore, Singapore 119391, Singapore; 5 NUS Graduate School-Integrative Sciences & Engineering Programme, National University of Singapore, Singapore 119077, Singapore

**Keywords:** autism spectrum disorder, autism, ASD, epigenetic, methylation, maternal immune activation, perinatal, microbiome

## Abstract

Autism spectrum disorder (ASD) is a complex neurological and developmental condition that occurs in approximately 1 in 100 children. ASD is a lifelong condition defined by difficulties with social communication, restricted interests, and repetitive behaviors, among other symptoms. Currently, we understand that there is no cure and the disorder can only be managed with occupational therapy alongside limited medical treatments. Reasons underlying the pathogenesis of ASD are still not well understood, but recent studies point to the influence of epigenetic dysregulation in ASD development, which opens up avenues to novel diagnosis and treatment options. In this review, we summarize recent findings and emerging therapeutics for ASD, with a focus on implications of epigenetic regulatory pathways and factors. We expound the implications of these findings to enable preventive measurements for mothers to reduce the impact of ASD at birth, non-invasive diagnostic tests for early detection, and personalized medicine management. Finally, we discuss several critical issues to be addressed and future directions of this important research field.

## 1. Introduction

### 1.1. Autism Spectrum Disorder

ASD is a complex neurological and developmental condition that is defined by core symptoms of social communication difficulties, restricted interests, and repetitive behaviors [1]. Individuals with ASD have difficulty expressing reciprocal emotions or sharing interests and often struggle with understanding social cues. Furthermore, behaviors like rigid routines, hyper-focus on niche subjects, sensory difficulties, and stereotypical movements such as hand flapping are also common. ASD patients manifest a spectrum of these phenotypes, and not all individuals present with the same symptoms to similar extents.

### 1.2. Current Therapeutics

ASD is a lifelong condition that is not ‘curable’. The management of ASD often involves a cross-disciplinary approach combining educational interventions, behavioral, speech-language, and occupational/physical therapies, alongside medical treatments [2]. One behavioral intervention is applied behavior analysis (ABA), which aims to promote or prevent certain behaviors using reinforcement or extinction strategies, having a proven track record to improve prosocial behaviors in ASD children over 70 years of use [3]. ABA is most effective before the age of 5. Further, 20–50% of children in ABA-modeled early intervention programs achieve average IQ, enabling placement into general education settings [4]. ASD children are also often enrolled into speech therapy, during which speech-language pathologists design treatment strategies to encourage joint attention (e.g., pointing at an object to indicate interest), social reciprocity (e.g., turn-taking during play), and language processing skills (e.g., understanding the use of symbols such as letters and words to represent spoken language) [5]. Many children with ASD also have sensorimotor impairments, which can affect their performance in academic settings, and this can be modulated by occupational therapy (OT) to significantly augment sensory processing and refine motor difficulties to increase functionality and independent daily living skills. Gross motor deficits can also be improved using physical therapy (PT) in ASD children.

Psychopharmacological treatments are also available, albeit to target maladaptive behaviors (including stereotyped behaviors, hyperactivity, and emotional lability) rather than addressing the core symptoms of ASD. Risperidone is a USA Food Drug Association (FDA)-approved antipsychotic dopamine antagonist for treating irritability in ASD. The usage of risperidone was reported to improve self-injury, aggression, and agitation in 70% of children and adolescents, with a placebo response rate of 11.5% [6]. Aripiprazole is the second antipsychotic medication approved by the FDA and is a partial dopamine (D2) agonist that modulates dopamine release. In two large-scale randomized, placebo-controlled studies, ASD children aged 6–17 demonstrated significantly lowered scores on two autism screening tests (Autistic Behavior Checklist and Clinical Global Impression Scale) following aripiprazole treatment [7].

### 1.3. Emerging Therapeutics

Thus far, one overarching cause that underlies ASD has not been found as the disorder is associated with heterogeneity, and the diverse variation among individuals affected by ASD has precluded the discovery of clear diagnostic biomarkers to promote effective treatments of core ASD symptoms. At best, current diagnostics such as the Autism Diagnostic Interview–Revised (ADI-R) and Autism Diagnostic Observation Schedule (ADOS) can only employ subjective clinical assessments of child behavior and parent–child interactions. Such suboptimal measures have led to delayed diagnose and even misdiagnoses, particularly for high-functioning autism [8,9]. In fact, mathematical models conclude that ~80% females remain undiagnosed even at age 18, leading to delayed access to resources which often results in severe mental health consequences [10]. Recent research, however, increasingly shows an appreciation of modulating genetic contributors by environmental factors via epigenetic pathways, positing the targeting of epigenetic mechanisms as a promising treatment avenue for ASD. Furthermore, epigenetic indicators are emerging as novel biomarkers to potentially improve early diagnoses of ASD. Here, we will review these recent advances, which have also led to new understandings of ASD pathogenesis.

## 2. Maternal Interventions

With the belief that ASD may start developing in the fetal stage during pregnancy, or even before that, attention is now cast to managing maternal conditions, including, for example, the gut microbiome with nutrients, which are shown to decrease the risk of ASD via the modulation of epigenetic makeup. We will discuss some of these findings here:

### 2.1. The Gut Microbiome and the Gut–Brain Axis

The relationship between the gut microbiome and the brain is facilitated by a bidirectional connection called the gut–brain axis [11]. The gut–brain axis is not only a physiological connection between the hypothalamus of the brain and the large intestine, but also serves as a network that interacts with the immune and endocrine system through the exchange of neurotransmitters, metabolites, and cytokines [12]. This connection is mainly facilitated by the vagus nerve, which regulates digestion, heart rate, and the immune system as part of the parasympathetic nervous system [13].

**Figure 1:**
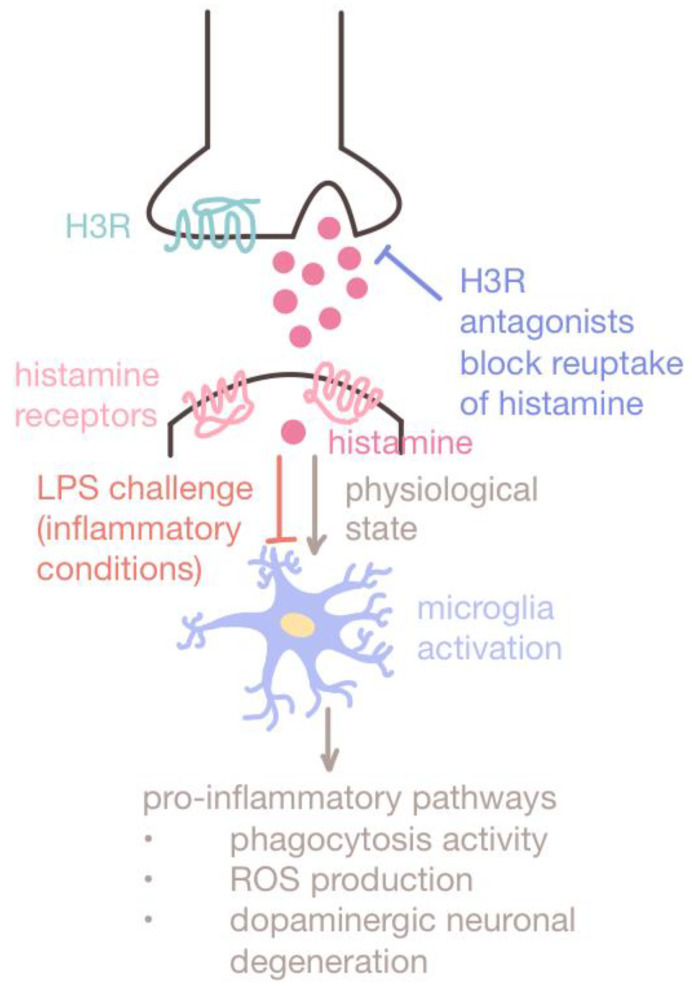
Dual role of histamine. Histamine takes part in both inflammatory and regulatory pathways. Multiple studies have proven that histamine plays a protective role under lipopolysaccharide (LPS) challenges or inflammatory conditions. The use of H3R antagonists to block the reuptake of histamine by H3R has been shown to enhance histamine release and inhibit chronic microglia activation. Under such inflammatory conditions, histamine protects dopaminergic neurons from degeneration, enhancing alertness and memory in VPA-exposed mouse models. Thus, histamine antagonists such as ciproxifan are promising therapeutics for ameliorating ASD phenotypes in MIA-exposed offspring.

From as early as the prenatal period, environmental factors such as microbes in the placenta, amniotic fluid, and blood of the umbilical cord influence microbiome development in the fetus [14]. Breastfeeding, skin contact, and vaginal delivery determine the inheritance of the gut microbiome from the mother to infants [15]. Research has shown that unbalanced maternal nutritional intake can increase the risk of neurodevelopmental disorders such as ASD, with alterations in the gut microbiota being associated with children diagnosed with ASD as compared to neurotypical controls [16]. Such changes in gut microbial diversity can compromise the integrity of the epithelial barrier, leading to ‘leaky gut’ or dysbiosis, in which increased intestinal permeability permits the passage of bacteria, toxins, and metabolites, thereby causing immune activation and intestinal disorders in the fetus [17]. The increased presence of the lipopolysaccharide (LPS) component of the bacterial outer membrane results in pro-inflammatory cytokines being released across the blood–brain barrier to induce central nervous system (CNS) inflammation. These changes can indirectly affect the fetal immune system and further induce cortisol release via the hypothalamic–pituitary–adrenal (HPA) axis to result in oxidative stress (Figure 1). Further evidence supports the association of unbalanced dietary patterns in mothers prior to conception with an increased risk of ASD in offspring [18], thus positioning maternal dietary interventions as promising preventative measures for reducing the risk of ASD and mitigating behavioral deficits in offspring.

Metabolites and the fermented products made by gut microbiota have been observed to induce epigenetic changes in the digestive tract and brain, leading to the proposal that these compounds can act as epigenetic agents, rather than simply as neurotransmitters, in the ASD context [19]. Decreased levels of *Bacteriodetes* bacteria are associated with the gut microbiota of ASD patients; treatments that augment members of this microbiota phylum via probiotic and dietary modulation benefit ASD intervention [20,21,22]. It was noted that *Bacteriodetes* microbes metabolize 3-oxolithocholic acid in bile salt to isoallolithocholic acid, which can enhance the differentiation of anti-inflammatory regulatory T (Treg) cells via epigenetic modulation [23]. Interestingly, recent research has shown that the immunoregulatory induction of maternal Treg cells can prevent autism-like behaviors in adult progenies [24], raising the need for bile salt metabolic regulation in mothers to be explored more to reduce ASD risk in offspring.

### 2.2. Maternal Nutrition

#### 2.2.1. Fatty Acid Intake

Polyunsaturated fatty acids (PUFAs) are immunomodulators that possess anti-inflammatory properties in the brain via microglia regulation [25]. PUFAs inhibit the p38MAPK inflammatory pathway, which modulates microglia activity, thus inhibiting cytokine release and neuroinflammation. The reduced consumption of foods high in PUFAs, like oily fish (such as anchovies, herring, mackerel, salmon, and sardines), nuts and seeds, and tofu/soybeans [26], and imbalanced maternal diets resulting in the reduced ingestion of omega-3 and two essential fatty acids (linoleic and alpha-linolenic fatty acids) are associated with increased ASD risk [27]. Among the PUFAs, n-3 PUFAs are often linked with anti-inflammatory properties, whereas n-6 PUFAs promote inflammation. Increasing n-3 PUFA consumption to over 450 g compared to the recommended 340 g a week in pregnant women is correlated with a lower incidence of neurophysiological dysfunctions in offspring [28]; however, the intake of less than 340 g of n-3 PUFAs per week by pregnant women is associated with deficits in prosocial behavior and motor deficits in the offspring [29]. These observations lend support to the beneficiary effects of the recommendation of the consumption of 250 mg per day of eicosapentaenoic (EPA) and docosahexaenoic acids (DHA) for pregnant women by the US Department of Health and Human Services [30].

Conversely, excessive free fatty acid intake—defined as >35% calories from fat—is a risk factor for ASD. In a study with female mice on a 60% fat diet for 8 weeks, their offspring showed ASD-like symptoms of low sociability, repetitive behaviors, and symptoms of anxiety [31]. Future studies should aim at investigating the ideal levels and types of fatty acids for consumption by pregnant women.

#### 2.2.2. Folic Acid Supplementation

Folic acid or folate is a soluble B vitamin that is important for epigenetic modulation [32], immune regulation [33], and cell repair in the CNS [34]. Its overall beneficial effects have led Canada and the US to mandate the fortification of grain-based food products with folic acid since 1998 [35]. Currently, the World Health Organization (WHO) prescribes at least 400–800 µg of folate per day [36].

Because folate is not synthesized by the human body, maternal dietary folate is the sole source of folate for the developing fetus [37]. Consistently, folic acid supplementation during pregnancy, especially before and in early stages, is associated with a 43% reduced risk of ASD pathogenesis [38]. On the contrary, maternal folic acid deficiency leads to autism-like traits in rats, affecting learning and memory probably via the suppression of neuronal migration and synaptogenesis in the cerebellum and hippocampus [39,40]. Reduced folic acid and methionine lead to a compromised supply of methyl moiety for epigenetic and metabolic regulation, in turn aggravating apoptosis and DNA damage in cultured hippocampal neurons [40,41]. Even though oral treatments of the methyl donor SAM ameliorated ASD-associated deficits in valproic acid (VPA)-exposed mice by reversing epigenetic changes, there has been limited success thus far in the development of preventative agents similar to SAM for the clinical management of ASD in humans.

#### 2.2.3. Choline Supplementation

Choline is a methyl group donor that regulates the gene expression involved with synaptic plasticity and is greatly beneficial for learning and memory. Gestational choline can impact the methylation of promoter DNA to regulate the expression of key epigenetic enzymes in brain cells [42,43].

Although choline is produced endogenously in the human liver, the amount produced is insufficient for healthy bodily function and consequently needs to be obtained through dietary supplementation by ingesting beef, eggs, chicken, fish, nuts, and legumes, among others. According to the Office of Dietary Supplements, pregnant women should increase choline intake from 425 to 450 mg/day. Children of mothers who took daily choline supplements showed a 5 unit increase in receptive communication and a 3.5 unit increase in expressive communication on the Bayley-III scales [44]. Thus, choline holds promise as a prenatal supplement for improving social communication deficits. However, unlike folic acid, there are currently no mandates for choline in Canada or the US.

## 3. Interventions on ASD Patients

### 3.1. Pharmacological Intervention

#### 3.1.1. Maternal Immune Activation Therapeutics

Maternal immune activation (MIA), or the initiation of inflammatory pathways during pregnancy, has been associated with the pathogenesis of neurodevelopmental disorders, including ASD [45,46,47]. MIA can be induced by a variety of factors such as maternal gestational diabetes, obesity, nutritional deficit, infection, or general stress [48], facilitated by bidirectional communication between the immune and endocrine systems. Immune cells are enriched with hormonal receptors on their cell surfaces that enable them to be highly sensitive to perturbation in endocrine homeostasis [49].

**Figure 2:**
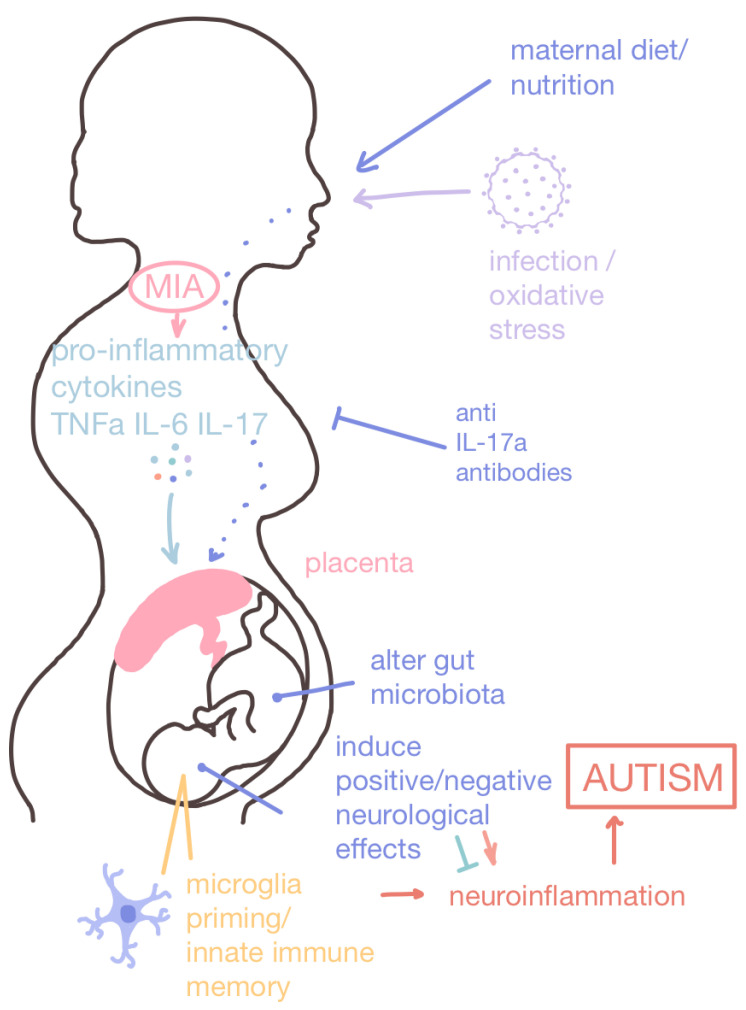
Maternal immune activation (MIA) and nutritional intake for the developing baby. Maternal immune activation (MIA) can be induced by a number of factors, including obesity, diabetes, infection, and oxidative stress. MIA activates microglia which release pro-inflammatory cytokines that recruit immune cells and amplify the inflammatory response. The placenta provides oxygen and nutrients to the developing baby through the umbilical cord while also removing waste and filtering out potentially harmful teratogenic chemicals. The mother’s cytokines can induce an inflammatory response in the developing baby and, if they penetrate the blood–brain barrier, induce neuroinflammation. Microglia can also be primed in a process called innate immune memory, which can lead to long-term deficits in microglia function. Maternal nutritional intake also has a direct impact on offspring health, as a high-fat diet is a risk factor for ASD pathogenesis. Contrarily, prenatal nutritional interventions can reduce the risk of offspring developing ASD or ameliorate ASD-associated symptoms.

Upon the induction of the maternal immune system, activated microglia releases inflammatory cytokines to amplify the inflammatory response and recruit immune cells to the site of inflammation [50]. In the event that maternal cytokines traverse the blood–brain barrier of the fetus, a neuroinflammation cascade is initiated, leading to neuronal dysfunction, which subsequently results in neurodevelopmental abnormalities (Figure 2) [47]. Microglia are the primary immune cells in the CNS responsible for synaptic maturation and the maintenance of neural circuits. MIA reprograms microglia in a phenomenon known as trained immunity (otherwise known as innate immune memory) to enhance immune responses or suppress immune pathways to confer immune tolerance [51] via epigenetic modulations involving histone modifications, DNA methylation, chromatin remodeling, and miRNAs [52]. In mouse models, MIA induced behavioral deficits, such as impaired vocalization, that lead to social interaction and inhibitory control difficulties and amphetamine hypersensitivity analogous to repetitive locomotion [53,54,55].

The following interventions by histamine receptor antagonists and IL-17a antibodies have been shown to reduce the chronic effects of neuroinflammation via targeting components of the MIA pathway to ameliorate social and behavioral difficulties in MIA-exposed offspring:

##### Histamine Receptor Antagonists

Histamine plays a significant regulatory role in the immune response as well as in sensory and motor functions, cognition, attention, and social recognition memory [56,57]. Histamine acts by binding to histamine receptors (H1R, H2R, H3R, and H4R) and exhibits both inflammatory and regulatory effects depending on the environmental context (Figure 1). While histamine is involved with pro-inflammatory pathways, when subject to inflammatory conditions (such as treatment with LPS inflammogen), histamine inhibits chronic microglia activation and exhibits downregulatory effects [58]. The LPS-induced experimental inflammatory conditions may be analogous to MIA conditions.

Histamine-targeting therapeutics may be used to downregulate neuroinflammation and reduce the risk of MIA-exposed ASD offspring. Most studies have chosen to target H3R, a presynaptic autoreceptor that regulates dopamine, acetylcholine, and histamine release via negative feedback, which has also been associated with other neurologic disorders such as Alzheimer’s disease and attention deficit hyperactive disorder (ADHD) [59,60]. The inhibition of H3R via H3R antagonists enhances histamine release, thereby suppressing the neuroinflammatory effects of microglia and preventing dopaminergic neuronal degeneration [58,61]. H3R antagonists such as ciproxifan have also been effective in enhancing alertness and memory [60], alleviating repetitive behavior in VPA-exposed mice [62], and correcting aberrant histamine-modulated neural activity in the cortico-basal ganglia circuitry [63,64] (Figure 1). Other studies have demonstrated that treatment with H1R and H4R antagonists reduces the production of pro-inflammatory cytokine IL-6 [65,66]. Additionally, histamine reversed cognitive decline in mice exposed to inflammatory conditions by enhancing neuronal proliferation in the hippocampus of adult mice [67].

Taken together, histamine antagonists might be effective in rescuing autism-associated behavior deficits induced by MIA in utero through histamine modulation. Although there is limited clinical evidence to support the effectiveness of H1R and H4R antagonists in trials involving targeting allergic and inflammatory diseases [68], these new findings point to the potential shown by histamine and histamine receptor antagonists that can be explored in future therapeutic strategies for ASD.

##### IL-17a Antibodies

IL-17a is a cytokine involved in the inflammatory pathway of MIA that triggers the activation of pro-inflammatory signals, which is found to be expressed at high levels in the blood of patients with ASD, despite being rarely expressed in the human brain except under inflammatory conditions [69]. In fact, the direct injection of IL-17a into the fetal lateral ventricles of mice resulted in ASD-like behaviors and MIA-induced abnormal cortical development, suggesting that neuroinflammation plays a primary role in ASD pathogenesis. Progenies of the ASD mouse model are prone to develop inflammation in the gut and were found to be associated with exposure to maternal inflammation that may partly arise from the IL-17a-dependent alteration of maternal gut microbiota, leading to postnatal changes to the chromatin makeup of naïve CD4+ T cells in the offspring [70]. Consistent with the connection of IL-17a-induced inflammation in ASD, the administration of anti-IL-17a-monoclonal antibodies to block the activation of inflammatory pathways associated with IL-17a constitutes a promising therapeutic avenue for ASD (Figure 2).

A study involved pretreating poly(I:C)-injected mice mothers with anti-IL-17a-monoclonal antibodies to induce MIA via cytokine release, was reported to partially alleviate abnormal social interaction difficulties and repetitive behaviors and preserve normal cortical development in MIA offspring [71]. Currently, secukinumab and ixekizumab are two FDA-approved IL-17a antagonists for psoriatic arthritis treatment [72,73]. Psoriasis occurrence in mothers was identified as a risk factor for MIA induction and thus would also be potentially linked to autism [74]. As such, future studies are needed to explore the causal relationship between MIA and ASD via IL-17a signaling to infer the effectiveness of secukinumab and ixekizumab for ASD treatments.

#### 3.1.2. CBD-Rich Cannabis

Cannabis has been used to treat epilepsy in the United States owing to therapeutic benefits such as improving sleep and the alleviation of anxiety and chronic pain [75]. Recent evidence points to the potential of cannabidiol (CBD)-rich cannabis in improving core symptoms of ASD [76]. CBD is an active ingredient in cannabis that exhibits “no effects indicative of any abuse or dependence potential”, as cited by WHO, in contrast to tetrahydrocannabinol (THC), the primary psychoactive component in cannabis that causes the ‘high’ in marijuana. However, its hallucinogenic effects can be masked at low concentrations.

In a randomized, double-blind, placebo-controlled trial, 150 participants with ASD were assigned either an oral placebo, a whole plant cannabis extract containing CBD and THC at 20:1 ratio, or pure CBD and pure THC at the same ratio and concentration for a 12 week period. Results indicated that the whole plant extract with CBD and THC at 20:1 ratio improved disruptive behaviors associated with ASD [77]. Another study that includes 33 children diagnosed with ASD received an average daily CBD-enriched cannabis dose of 0.7 mg/kg/day for an average of 6.5 months. The children exhibited decreased behavioral problems, an increase in expressive language, and improvements in cognition and social interaction. Additional benefits of CBD-enriched cannabis include a reduction in other prescription drugs and a reduction in the frequency of seizures for participants with comorbid epilepsy [78]. Other case studies and open-label studies, albeit without the necessary experimental controls, also reported significant improvements in behavioral assessments using ADOS-2, a social responsiveness scale (SRS), and vineland adaptive behavior scales (VABSs) [79], in addition to enhancements of focusing and learning capabilities [80].

The use of CBD is not without health risks as it induces a heightened risk of liver damage, adverse effects on the male reproductive system, and potential drug interactions [81]. Additionally, participants in the Bilge 2021 study reported mild side effects of restlessness, and some participants experienced generalized seizures even after cessation of treatment [78]. There are several potential mechanisms by which CBD may exhibit regulatory effects on the endocannabinoid system (ECS), which controls critical bodily functions such as appetite, memory, and motor responses. CBD is a non-competitive negative allosteric modulator of the cannabinoid receptor 1 (CB1), an endocannabinoid receptor, in the hippocampus, basal ganglia, basolateral amygdala, hypothalamus, and cerebellum [82]. Although exact mechanisms are still unclear, CBD may inhibit endocannabinoid signaling and its effects on synaptic plasticity through negative allosteric modulation [83], while the enhancement of vasopressin and oxytocin release by CBD and other epigenetic modulations have also been proposed [78,84].

Overall, CBD-enriched cannabis is an incredibly promising therapeutic that targets the core negative symptoms of ASD. Its side effects and the current limited understanding of its drug interactions and properties, however, pose significant issues for the immediate application, requiring further research to optimize its effectiveness in ASD.

#### 3.1.3. Oxytocin

Oxytocin (OXT) is a nonapeptide produced mainly in the hypothalamus and projected to various reward and social brain circuits. Oxytocin and the oxytocin receptor (OXTR) are programmed by early life experience and linked to social behaviors such as eye gaze and face perception [85]. The hypermethylation of specific 5′-C-phosphate-G-3′ (CpG) dinucleotides is detected in CpG islands previously shown to affect OXTR gene expression in ASD individuals that are correlated with aberrant oxytocinergic signaling and social interaction deficits [86].

Oxytocin counterbalances the cortisol released by the HPA axis to regulate stress response. The quantification of salivary OXT levels in ASD children revealed a ‘trait dependency’ to OXT levels—in particular, a non-stress-reactive ‘state-dependent’ level of OXT was altered in children with ASD. The diminished levels of ‘trait-dependent’ OXT observed in children with ASD is indicative of dysfunctional coupling between OXT and cortisol, which may potentially underlie the deficiency demonstrated by children with ASD in the protective mechanisms that counteract social stressors [87].

Multiple randomized, double-blinded, placebo-controlled studies involving the chronic administration of intranasal OXT was conducted, albeit presenting with mixed outcomes. In one study on the Shank3-deficient ASD mouse model, the chronic administration of OXT prevented social deficits after 2 weeks of treatment but led to social ambivalence beyond that. Treated animals exhibited reduced interest in their surroundings and repetitive self-injurious behavior 4 weeks after the termination of treatment, possibly due to the downregulation of the OXTR over time [88]. On the other hand, in a human study, four-week chronic OXT administration stimulated the oxytocinergic system in children with ASD, leading to heightened OXT levels and reduced methylated sites in the OXTR [89]. Yet, some other studies have reported no overall benefit of OXT treatment for 3–12 years old children.

A recent promising study, however, demonstrated that effects on gene regulation by chronic OXT administration may be dose- and frequency-dependent, showing most encouraging outcomes when used in conjunction with social behavioral therapy. In this study, intranasal OXT was administered every other day and paired with 30 min of positive social interaction for a period of 6 weeks. Young children with ASD showed significant improvements not only in both subjective measures of social behaviors (using SRS-2 and ADOS-2) but also in two eye-tracking paradigms, with no negative impacts reported even after 6 months following treatment [90].

Due to the heavy involvement of the oxytocinergic system in regulating social behaviors and learning, future research directed at variations in dosage, frequency, age, and co-treatment with behavioral therapies will be important in optimizing OXT therapeutics.

#### 3.1.4. Histone Modifiers

Recent evidence shows that DNA methylation and histone modifications are linked to the etiology of ASD. In fact, many prominent ASD risk genes encode histone methyltransferases and demethylases, such as euchromatic histone lysine methyltransferase 1 (EHMT1), lysine methyltransferase 2C (KMT2C), and lysine-specific demethylase 1 (LSD1) [91]. Individuals with ASD exhibit dysregulated neuronal histone H3 lysine 4 (H3K4) methylation as well as upregulated histone deacetylase 4 (HDAC4) expression in the prefrontal cortex [92].

Histone deacetylase inhibitors are promising therapeutics that have been shown to correct histone acetylation levels and ameliorate disruptive behaviors often associated with ASD. Sodium butyrate, a HDAC inhibitor, improves sociability in Black and Tan BRachyury (BTBR) T^+^ltpr3^tf^/J mouse models through transcriptional changes in excitatory and inhibitory genes in the prefrontal cortex [92]. The combined treatment of HDAC and LSD1 inhibitors also downplayed aggression in Shank-3 mice via the transcriptional upregulation of the N-methyl-D-aspartate (NMDA) receptor, which regulates cognition and emotion [93]. Romidepsin is one such HDAC modifier that acts on similar mechanisms to improve sociability in ASD. Brief treatment with a low dose of romidepsin on Shank-3 mice led to the prolonged restoration of social behaviors for three weeks (equivalent to several years in humans) [94]. As an FDA-approved anti-cancer drug, romidepsin holds potential as a pharmacological treatment that corrects epigenetic abnormalities in ASD.

#### 3.1.5. DNA Methylation and Genomic Imprinting

Genomic imprinting is an epigenetic phenomenon in which one of the two alleles of an ‘imprinted’ genes becomes epigenetically repressed to confer monoallelic expression, often in an allele-specific manner that is dependent upon the parental origin of the allele [95,96]. Numerous neurodevelopmental genes inclusive of ASD candidate genes are among the imprinted genes exhibiting allele-specific expression [97,98]. These genes are observed to be differentially methylated on their DNA in ASD subjects relative to controls [99,100,101]. Some of these sites are enriched with CCCTC-binding factor (CTCF) binding motifs that suggest the formation of abnormal higher-order chromosomal conformation to underlie the manifestation of ASD phenotypes [99]. Studies have uncovered differentially methylated profiles in paternal sperm DNA that is linked with the risk of ASD at birth [102], and the usage of a psychoactive agent, which is also associated with the risk of ASD at birth, can alter DNA methylation patterns on maternally imprinted and ASD candidate genes in the sperm [103].

These findings thus point to a close connection between the deregulated DNA methylation of imprinted loci with the risk of ASD at birth and has motivated attempts to uncover critically imprinted domains of predictive value for ASD prognosis. One such loci is the 15q11-13 locus, which is also linked to Prader–Willi and Angelman syndromes [104,105]. 15q11-13 hosts clusters of ASD candidate genes that include the gamma aminobutyric acid A ((GABA)_A_) neurotransmitter receptor subunit genes reported to show monoallelic expression in ASD subjects and opposes biallelic expression in normal controls [105]. A recent genome-wide study of monoallelically expressed genes from ASD samples, however, revealed heterogeneity that confounds the link between parental imprinting and ASD risk [106]. A similar observation was made by another group at the distal 8q locus which hosts the *KCNK9* gene and a paternally imprinted non-coding *PEG13* coding sequence, where a clear correlation between genomic imprinting and ASD was not found [107]. Such complexity, in a way, is expected from the complicated interaction between multiple genetic loci to influence the development of ASD phenotypes, which can be further confounded by the involvement of specific environment stressors [108]. Hence, more in-depth genome-wide investigations are needed to dissect the intricate connection between imprinted genomes and specific ASD phenotypes to facilitate the future development of diagnostics and therapeutics.

#### 3.1.6. Epigenetic Interplay of Signal Transduction Regulation

The connection between signal transduction defects and ASD pathology has opened up the treatment possibility of pharmacologically targeting these pathways, including PI3K/AKT/mTOR (mTOR), Wnt/β-catenin (WNT), Notch, brain-derived neurotrophic factor (BDNF)-TrkB, sonic hedgehog, retinoic acid, bone morphogenetic protein (BMP), and MAPK/ERK pathways. Because many extensive reviews have been published on each [109,110,111,112,113,114,115,116], we will focus our discussion herein on recent discoveries in this topic.

##### mTOR

The dysregulation of mTOR is a central mechanism underlying synaptic abnormalities in ASD [117]. Epigenetic factors such as the histone demethylase KDM6B, the transcription co-factor and chromatin remodeler Zmiz1, and the *MSNP1AS* long non-coding RNA (lncRNA) have been reported to modulate mTOR activity. KDM6B, which demethylates H3K27me3, counteracts the repressive effects of the polycomb repressive complex 2 (PRC2) on the expression of the NMDA receptor genes by modulating mTOR regulation [118,119]. The activation of NMDA receptors reciprocally inhibits the hyperactivation of mTOR [120]. Zmiz1, an ASD-risk gene highly expressed in the neocortex, hippocampus, and cerebellum, binds to chromosomal regions packaged with open chromatin [121]. Zmiz1 mutations impair neuronal differentiation and synaptic gene expression and are linked to reduced mTOR activity in other contexts, such as melanogenesis [122]. Additionally, long non-coding RNA *MSNP1AS*, which is highly expressed in ASD patients, suppresses mTOR signaling by downregulating moesin protein expression, reducing neuronal viability, and impairing neurite outgrowth [123,124]. Targeting these factors holds treatment potential for ASD patients with dysregulated mTOR signaling.

Estrogen therapy is known to have a protective role against neurodevelopmental disorders and glutamate-induced neurotoxicity via the mTOR pathway [125]. Estrogen confers methylation on the GC-rich SP-1 promoter DNA motif [126] to repress the expression of the *Ephx2* gene which encodes epoxide hydrolase (sEH). sEH is an enzyme that converts epoxy fatty acids (EFAs) into pro-inflammatory vicinal diols, which are elevated in critical brain regions of ASD patients [127,128]. The inhibition of sEH using 1-trifluoromethoxyphenyl-3-(1-propionylpiperidin-4-yl) urea ameliorates cognitive defects in the offspring of the ASD mouse model [128]. This corroborates with another recent study that reported sEH-dependent alleviation of social deficits and fear responses in ASD mice occurs through the restoration of dysregulated mTOR-related pathways [129]. Collectively, these findings suggest that reducing sEH and restoring mTOR signaling could be achieved epigenetically through the estrogen-dependent DNA methylation of *Ephx2*.

In addition to epigenetic regulation, the direct targeting of mTOR has been explored as a therapeutic strategy for ASD. The mTOR inhibitor everolimus can reduce Tuberous Sclerosis Complex-associated renal angiomyolipomas, subependymal giant cell astrocytomas, and ASD-related behaviors [130]. Furthermore, the postnatal exposure of Wistar rats to VPA followed by acute treatment with the mTOR inhibitor rapamycin ameliorated social difficulties and abnormal neuronal excitability [131]. Natural bioactive compounds derived from plants have also demonstrated benefits in preclinical and clinical studies for ASD management [132]. One such compound, chrysophanol, extracted from *Rheum palmatum*, has been shown to improve memory, learning, and social interaction in ASD mice through the downregulation of the mTOR pathway [133].

##### WNT

WNT signaling—tightly regulated by epigenetic factors—plays significant roles in neurogenesis, synaptic plasticity, and neuroprotection [134,135,136]. Epigenetic regulators are instrumental in modulating this pathway. Chromatin remodelers such as CHD8 and ARID1B, along with histone demethylases KDM5C and KDM2B, suppress WNT by either inhibiting β-catenin and regulating the expression of other WNT pathway genes [110,137,138,139]. Mutations in these epigenetic regulators can lead to the hyperactivation of WNT signaling that alters dendritic and spinal morphology to underlie ASD-like behaviors, including circuit dysfunction and social deficits. Conversely, the histone acetyltransferase KAT6A activates the WNT pathway by promoting the transcription of the *RSPO2* gene, which encodes a WNT activator R-spondin 2 [140]. The inhibition of KAT6A downregulates WNT pathway genes that regulate excitatory synapse function, leading to reduced dendritic spine density in CA3 pyramidal neurons [140]. Similarly, the neuron-specific chromatin remodeler CHD5 mediates WNT activation by repressing WNT ligand Wnt5a via the transcription factor Six3 [141]. Wnt5a can either activate or suppress WNT depending on developmental context [142]. These findings highlight that the precise spatiotemporal control of WNT signaling is essential for normal neurodevelopment and synaptic function. However, they also raise challenges regarding pharmacological interventions targeting the WNT pathway for ASD treatment, as a careful balance of both WNT antagonists and agonists would be needed. Preliminary testing to understand patient-specific gene expression profiles and/or genomic sequencing to reveal genotypic nucleotide variants may assist the precise implementation of such strategies.

WNT signaling can also modulate ASD phenotypes via its connection with microtubule affinity-regulating kinase 2 (MARK2). A loss-of-function variant of *MARK2* is linked to developmental delays, communication difficulties, and movement difficulties in ASD individuals [143]. Recently, lithium, a mood stabilizer commonly prescribed for bipolar disorder [144], has been shown to reverse abnormal cellular phenotypes in MARK2-mutated neural progenitor cells. It also restored normal cortical layer formation in fetuses of lithium chloride-fed Mark2+/− pregnant mice [143]. Lithium mediates these effects via reactivating the downregulated WNT/β-catenin pathway in MARK2 mutants, in conjunction with enhancing BDNF expression via counteracting its repressive promoter DNA methylation [145]. BDNF regulates neurodevelopment and neuroplasticity, and altered BDNF levels are often observed in ASD individuals [146,147]. BDNF expression is stimulated by the activation of WNT/β-catenin signaling through the binding of the Wnt3a ligand in response to pain-induced WNT secretion, pointing to a link between WNT signaling and BDNF in the case of ASD [148]. Overall, these findings show the promise of lithium in ASD treatment.

##### Other Signaling Regulation

Epigenetic factors also influence other key signaling pathways implicated in ASD. The H3K36 methyltransferase ASH1L regulates BDNF-TrkB signaling, which regulates neuronal arborization during development [143]. Mutations in ASH1L disrupt the expression of the *NTRK2* gene that encodes the TrkB receptor, leading to impaired neurite outgrowth and synaptic deficits associated with ASD [149]. On the other hand, the hyperactivation of the Notch signaling pathway has been observed in the prefrontal cortex, hippocampus, and cerebellum of ASD models. This hyperactivation downregulates DNA methyltransferases DNMT3A and DNMT3B and impairs autophagy-related gene expression to contribute to ASD pathology [150].

**Figure 3:**
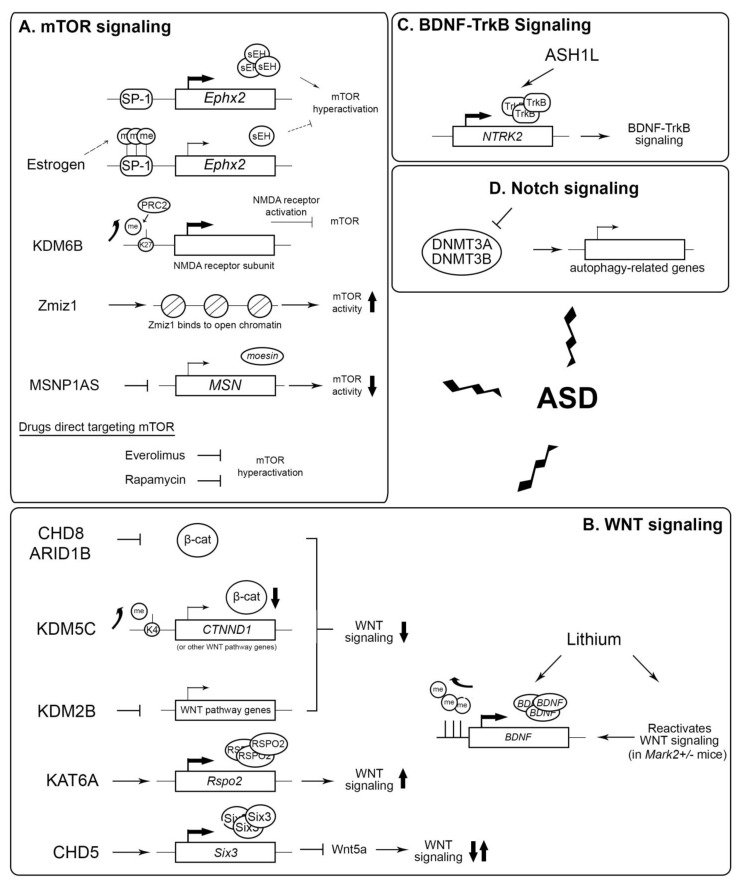
Potential epigenetic targets for the treatment of ASD. (**A**) Role of estrogen, KDM6B, Zmiz1, and MSNP1AS in the regulation of mTOR activity. Everolimus and rapamycin directly inhibit mTOR. K27: histone H3 lysine 27; me: methylation. (**B**) Effect of different epigenetic factors and lithium in modulating WNT signaling activity. K4: histone H3 lysine 4; β-cat: beta-catenin. (**C**) ASH1L activates the expression of *NTRK2,* which is important in BDNF-TrkB signaling. (**D**) The hyperactivation of Notch signaling inhibits DNA methyltransferases DNMT3A and DNMT3B expression, in turn reducing the expression of autophagy-related genes, leading to ASD. Zigzag arrows: the dysregulation of these signaling pathways lead to ASD. Up arrow: upregulation. Down arrow: downregulation.

Taken together, these recent findings have uncovered a mechanistic relationship between epigenetic dysregulation and several signaling pathways—mTOR, WNT, Notch, and BDNF-TrkB—in ASD development to pave the way for novel therapeutic developments to alleviate ASD symptoms (Figure 3).

### 3.2. Patient Nutrition

Several plausible dietary intervention regimens have been supported by recent research to benefit ASD patients:

#### 3.2.1. Ketogenic Diet

The low-carbohydrate and high-protein and -fat ketogenic diet (KD) has a number of advantages for ASD patients, as it can ease anti-social and repetitive behaviors by reducing pro-inflammatory cytokines and normalizing gut microbial composition [151]. KD significantly improved sociability, communication, and repetitive behaviors in the BTBR mouse model as well as in human studies and case reports. Following a 14-month ketogenic regimen, a 4-year-old girl with comorbid ASD and epilepsy showed an increase of 70 points on the IQ scale and in social skills on the Childhood Autism Rating Scale (CARS), placing her in the non-autistic range [152]. Additionally, a 9-year-old girl showed a 95% reduction in seizure frequency after the use of the KD [153].

#### 3.2.2. GF/CF Diet

The gluten-free/casein-free (GF/CF) diet eliminates all foods with gluten (wheat, barley, and rye) and casein (dairy products) in an attempt to reduce inflammation. Children with ASD on the GF/CF diet showed significant improvements in speech and behavior in CARS scores after 6 and 12 months of treatment [154]. In four out of five cases in a case–control study, improvements were so significant that children no longer met the diagnostic criteria for ASD [155]. Additionally, 25.6% of children with ASD have increased intestinal permeability compared to 2.3% of neurotypical children [156]. Gluten and casein release opioid peptides, which alter patterns of DNA methylation and gene expression and promote inflammation in the gastrointestinal (GI) tract [157]. These peptides can also permeate the intestinal membrane and cross the blood–brain barrier, targeting opioid receptors in the brain. The GF/CF diet eliminates the opioid peptides and is correlated with the restoration of gut microbiome diversity and intestinal barrier function. Long-term use of the GF/CF diet is effective in improving behavioral symptoms and GI inflammation.

#### 3.2.3. Probiotics

**Figure 4:**
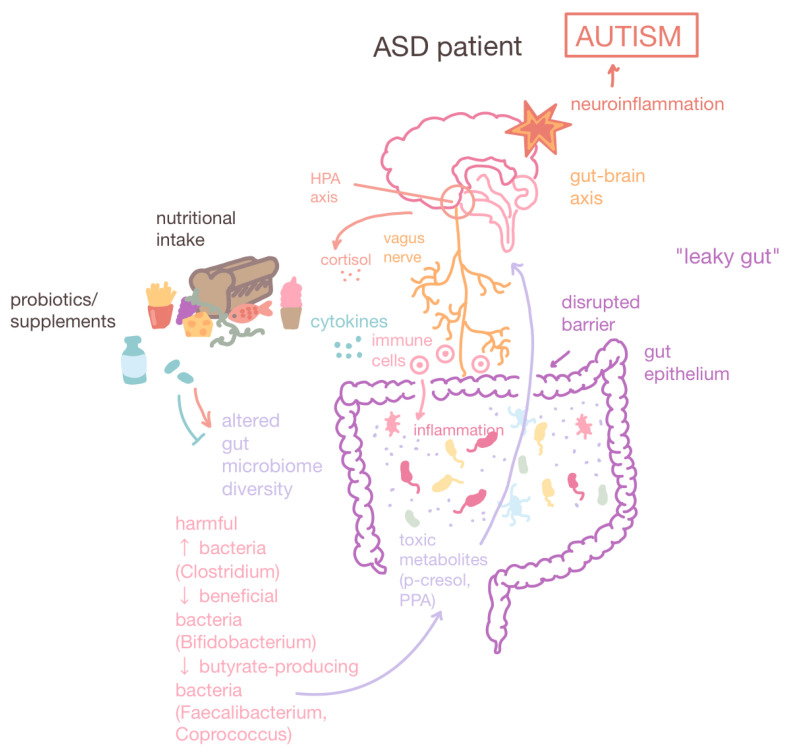
Gut–brain axis and altered gut microbiome compositions in ASD patients. The gut–brain axis is a bidirectional connection between the brain and the GI tract facilitated by the vagus nerve. ASD patients have altered gut microbiome compositions characterized by a decrease in diversity, an increase in harmful bacteria such as *Clostridium* which produce toxic metabolites such as PPA, and a decrease in beneficial bacteria (*Bifidobacterium)* and butyrate-producing bacteria (*Faecalibacterium* and *Coprococcus)*, which maintain intestinal barrier integrity. Leaky gut or gut dysbiosis is a state of increased intestinal permeability that leads to GI inflammation. Furthermore, leaky gut allows the greater passage of toxic metabolites which can potentially cross the blood–brain barrier and cause neuroinflammation. Dietary interventions and probiotics can correct gut microbial compositions, reducing GI inflammation and ameliorating ASD behavioral symptoms. Up arrow: increase. Down arrow: decrease.

Compared to neurotypical individuals, ASD patients are found to have significant alterations in gut microbial compositions. For example, ASD patients tend to have decreased levels of *Bacteroidetes* microbes, which facilitate polysaccharide digestion, and significantly higher levels of *Clostridium* species, which produce neurotoxins that are thought to be linked to the exacerbation of autistic symptoms (Figure 4). The *Clostridium* bacterial cells produce short-chain fatty acids (SCFAs), including propionic acid (PPA), which can promote behaviors associated with ASD including increased aggression and restricted behavior and cognition [158].

The probiotic supplementation of *Lactobacillus* and/or *Bifidobacterium* bacteria is associated with decreased intestinal barrier permeability and alleviated autistic behaviors. *Lactobacillus reuteri* has been found to alleviate social difficulties in people with ASD by improving attention, learning, and memory deficits, probably through the stimulation of the vagus nerve, resulting in subsequent OXT release [159]. Recent precision synbiotic therapeutics utilize nutritional surveys to determine the diet quality of ASD participants, collect and sequence fecal samples, and culture custom synbiotics using Floré’s proprietary formulation database and decision matrix analysis. The analyses revealed that 3 months of supplementation with personalized synbiotics was linked to an overall improvement in GI symptoms in 52% of participants and significant increases in probiotic species including *Bifidobacterium* and *L. reuteri* [160]. These studies support the use of probiotic treatment to modulate patient-specific GI and behavioral concerns to be a promising therapeutic for ASD symptoms.

## 4. Diagnostic Biomarkers for ASD

The absence of objective and reliable biomarkers for ASD patients often leads to delayed or even missed diagnoses. Screening and diagnosis rely on developmental monitoring by parents, medical professionals, and other caretakers. Current screening and diagnostic tools for ASD utilize the standardized diagnostic criteria provided by the Diagnostic and Statistical Manual of Mental Disorders (DSM-5); the most used include the Modified Checklist for Autism in Toddlers (M-CHAT), Ages and Stages Questionnaire (ASQ), Autism Diagnostic Interview-Revised (ADI-R), Autism Diagnostic Observation Schedule (ADOS), and Children Autism Rating Scale (CARS) [161,162]. According to the 2018 Autism and Developmental Disabilities Monitoring (ADDM) Network study, the age at first diagnosis ranged from 46 to 67 months [163]. Furthermore, ADDM Network data reveals a positive association between sagging eye syndrome (SES) and ASD diagnosis as well as persistent racial and ethnic disparities in ASD among low-SES children [164]. Additional studies comparing ASD symptoms of minority to non-minority ASD children suggest that this disparity could be explained by a decreased level of health literacy regarding early signs of atypical child development in lower SES parents and decreased resources for the screening and diagnosis of ASD [165]. Without biologically based markers for reliable diagnosis, ASD diagnosis can be subject to bias and can fail to identify children with ASD early enough during the critical period of development, thus predisposing them to suboptimal developmental outcomes [166]. In the following section, we will discuss emerging diagnostic biomarkers for ASD such as salivary microRNA (miRNA) tests, perinatal epigenetic signatures, glutathione ratios, and gut microbiome composition.

### 4.1. MicroRNA

MicroRNA (miRNA) is a single-stranded non-coding RNA with 20–22 nucleotides [167]. They are involved in almost all important biological processes, including the translation inhibition of target genes via imperfect base-pairing with the 3′ UTR of target mRNAs [168]. Due to its ability to link epigenetic contributions to neurodevelopment, the following miRNA subtypes have been identified as reliable biomarkers for ASD.

The miRNAs miR-21-3p and miR-21-5p are often overexpressed in the frontal cortex of ASD patients. The overexpression of miR-21-3p correlates with the inhibition of critical neuronal and synaptic genes such as *DLGAP1*, which decreased the expression of Protocadherin-19 (*PCDH19*), associated with cognitive impairment. The overexpression of miR-21-5p inhibits the OXTR translation that underlies social deficits. Furthermore, miR-103 and miR-146a have been found to be dysregulated in the lymphoblastoid cell lines of ASD patients. The dysregulation of miR-103 may be a contributing factor for abnormal fatty acid metabolism in ASD, and miR-146a defects may lead to impaired phagocytosis in immune responses. miR-146a has been identified as a promising diagnostic biomarker due to its association with neurogenesis, abnormal brain anatomy, and memory deficits [169].

MiRNAs can be collected and analyzed through multiple methods such as through the sampling of brain tissues and peripheral blood. Following the popularization of the administration of COVID-19 swab tests, samples that are easily obtained via non-invasive means, such as saliva samples, have drawn increased attention as an approach for infants. A pilot study of salivary miRNA, particularly miR-151a-3p measurements of 6-month infants, revealed the predictive value of neurodevelopmental delays at 18 months [170]. Another survey of 24 ASD children (compared to 21 non-ASD controls) undertaken in the United States identified 10 upregulated and 4 downregulated miRNAs in the salivary content of ASD. Subsequent target gene network analysis revealed the significant enrichment of genes in transcriptional activation and neuron and axon projections, preferentially in ASD-related subjects that are associated with neurodevelopment, among which are the Fragile X Mental Retardation (FMR) 1 and Forkhead Box Protein (FOXP) 2 [171]. A smaller Italian scale study involving five ASD and five controls produced more varied outcomes, although they also identified the enrichment of neurodevelopment target genes of the differentially expressed miRNAs. These include miR-199b-5p, shown to be upregulated in ASD children, which is involved in the regulation of *AUTS2* gene, associated with many neuropsychological disorders; miR-199a-3p, which regulates the cannabinoid receptor CB1 that underlies social rewards; and three other miRNAs that are associated with altered nicotinic receptor expression in the frontal, parietal cortex, and cerebellum, implicated in social cognition [172].

Salivary miRNA tests are effectively non-invasive screening and diagnostic approaches that show potential in allowing easy scale-up for improving ASD detection to facilitate more effective early intervention. However, current knowledge still falls short of arriving at a relative unanimous panel of diagnostic miRNAs for clinical application. A major reason stems from published papers involving small sample sizes that inevitably result in a lack of specificity and higher variation. More future clinical research, particularly that involves multi-centers with larger patient population, is thus needed.

### 4.2. Perinatal Epigenetic Signatures

Perinatal imbalance in the metabolite contents of placenta versus umbilical cord blood, as well as dysregulated epigenetic signatures such as DNA methylation profiles, are correlated with ASD development in offspring. In a study analyzing maternal mid-gestation (MMG) plasma at 18 weeks of gestation and umbilical cord blood plasma samples at birth, children that developed ASD later in their development exhibited metabolomic dysregulation, which is an accumulation of methionine levels in ASD children, among other metabolites [173], pointing to folate metabolism imbalances potentially diverting flux away from the formation of the S-adenosyl methionine (SAM) cofactor, which is essential for the methylation reactions that underpin epigenetic regulations [41]. This observation supports the beneficial effect of dietary folate supplementation to increase SAM production in women before conception and early gestation [174]. Interestingly, the use of VPA to treat seizures by mothers in the first trimester increased the risk of ASD at birth [175]. VPA can inhibit the enzymatic activity of histone deacetylases, which maintain epigenetic silencing at constitutive heterochromatic regions of the genome in conjunction with histone methylation that entails SAM [41,176].

Metabolic imbalances are correlated with membrane disruption, neurological dysfunction, and inflammation [173]. The imbalanced ratio between arachidonic acid and docosahexaenoic acid (AA/DHA) occurs in cord blood of mothers of ASD offspring, which was suggestive of the occurrence of inflammation. AA is an n-6 PUFA that serves as a precursor of pro-inflammatory eicosanoids, while DHA, an n-3 PUFA is involved in anti-inflammatory responses [177]. Furthermore, ASD children also show reduced phosphatidylcholine (PC), which is important for membrane integrity and transport in MMG plasma. This study also reported dysregulated glutamate and glutamine biosynthesis that caused elevated plasma glutamate and reduced plasma glutamine in MMG plasma. Glutamate plays a critical role in neuronal migration and plasticity by being an excitatory neurotransmitter, and an excess of glutamate is expected to result in the excitotoxicity-induced apoptosis of neurons. On the other hand, glutamine is a precursor for the synthesis of not only glutamate but also the inhibitory GABA neurotransmitter. As such, an imbalanced glutamate/glutamine ratio would thus result in impaired neurotransmission and neurotoxicity.

These metabolic imbalances can possibly also bear an epigenetic connation. An ASD-unrelated study of neuronal dysregulation via Niemann-Pick type C1 (NPC1) deficiency revealed that imbalanced glutamine/glutamate metabolism accompanying folate metabolism disturbances in cerebellum neurons is correlated with widespread DNA methylation defects in the genome [178]. A similar mechanistic disruption possibly also occurs in ASD.

The human placenta has a distinct DNA methylation profile, with extensive partially methylated domains. Differential levels of methylation in the DNA of placenta and peripheral cord blood tissues have been reported in children with ASD. For example, the *AUTS2* gene is hypermethylated in the placenta of children who later develop ASD, indicative of impaired dendrite and axon elongation in neuronal migration [179]. Additionally, multiple *WNT* genes are hypermethylated in the ASD placenta. Of these, *WNT1* and *WNT2* are critical for embryonic brain development, while *WNT7B* regulates the blood–brain barrier and CNS angiogenesis. X-linked epigenetic dysregulation has also been recorded in the cord blood of newborns who later are diagnosed with ASD, offering a possible explanation for the male bias in ASD as females are protected by the presence of a second X chromosome [180].

Currently the average youngest age for ASD diagnosis is 4–5 years old [181]. Thus more markers need to be discovered to permit even earlier diagnoses or even prognoses. Epigenetic marks such as histone modification hold promise in this aspect. A recent longitudinal study surveyed epigenetic profiles of mononuclear cells in umbilical cord blood extracted from 41 ASD and 63 developmentally delayed newborns, compared with 2519 normal children, with follow-ups until 3 years of age. This study identified higher histone H3 lysine 9 trimethylation (H3K9me3) levels at the promoter of the gene encoding retinoic acid receptor (RAR)-related orphan receptor alpha (*RORA*). Reduced *RORA* expression was detected alongside that of superoxide dismutase and G protein-coupled estrogen receptor-1 genes, as well as upregulated progesterone receptor expression. The levels of H3K27me2, H3K27me3, H3K9me2, and DNA methylation on the *RORA* promoter were, however, not significantly different, pointing to the efficacy of the co-analysis of specific epigenetic markers with the gene expression of key neural developmental genes as predictors of ASD occurrence from as young as newborn babies [182].

### 4.3. Glutathione

The anti-oxidant glutathione (GSH) has emerged as a promising ASD biomarker. Reactive oxygen species (ROS) arising from oxidative stresses, especially under interrupted mitochondrial oxidative phosphorylation, trigger apoptosis that is counteracted by GSH. Persistent ROS presence under high oxidative stress exposure can lead to more damage to mitochondrial electron transport chain complexes to further increase ROS production in a positive feedback manner, which eventually depletes GSH. A decreased GSH–oxidized glutathione ratio that signals prolonged oxidative stress is consistently observed in ASD individuals, as reported by multiple meta-analyses [183,184,185,186].

The human brain is particularly vulnerable to oxidative stress due to its high content of oxidizable PUFAs and redox-active metals such as copper and iron [187]. Children are more vulnerable than adults owing to their naturally low GSH levels during the perinatal period, which coincides with the time of development of ASD. Such oxidative stress in the brain can induce reversible and irreversible post-translational modifications of proteins, deficits in lipid metabolism, and the accumulation of toxins. In fact, the prediction of ASD pathogenesis risk could reach 90% accuracy based on trans-sulphuration and trans-methylation metabolites, such as GSH, that are collected during the third trimester [188]. SWI/SNF chromatin remodeler ARID1A-deficient cancer cells show vulnerability towards oxidative stress that arises from GSH depletion due to the reduced expression of the SLC7A11 cysteine transporter, which also regulates folate metabolism [41,189]. It is possible that chromatin remodeling defects resulting in similar metabolic compromises may act synergistically with oxidative stress and/or GSH depletion to contribute to ASD pathogenesis.

### 4.4. Gut Microbiome Composition

Multiple metabolites in the gut microbiome are potential non-invasive diagnostic markers for ASD. For reasons not yet clarified, ASD individuals tend to host microbiota with an increased number of harmful bacteria (e.g., *Clostridium* species) and the lower enrichment of beneficial bacteria (e.g., *Bifidobacterium* species). Other bacteria such as *Blautia*, *Eubacterium hallii*, *Subdoligranulum*, *Coprococcus*, and *Ruminococcus* species are also reported to be biomarkers of ASD in 3–6 year old children, with an area under curve (AUC) of 0.88 [190].

As leaky gut is a risk factor for neuroinflammation, the lactulose–mannitol ratio has been employed to be a diagnostic biomarker for intestinal permeation [191]. Lactulose is a large molecule that permeates when the intestinal barrier is compromised, while mannitol is a small molecule that permeates under regular conditions. Studies have also found that depleted amounts of butyrate-producing bacteria in ASD individuals, such as *Faecalibacterium* and *Coprococcus*, can counteract the leakiness of the gut (Figure 4) [192,193,194].

A recent study by the Chinese University of Hong Kong designed a panel of 31 multi-kingdom markers including bacteria, viruses, fungi, and other microorganisms and showed a promising AUC of 0.91 for ASD diagnosis. This panel was validated across multiple age groups ranging from 1 to 13 years old, all showing relatively high AUCs of 0.87–0.91 [195]. Although no causal relationship has been implicated between these biomarkers and ASD pathogenesis, significant correlations have been drawn that can potentially be applied as non-invasive diagnostic biomarkers of ASD.

## 5. Conclusions

This review summarizes recent discoveries in ASD, particularly the connection to epigenetic regulation. The appreciation of maternal immune activation and microbiota influence positioned nutritional modulation as a preventive intervention for mothers, possibly to reduce the incidence of ASD childbirth. Pharmacological management, on the other hand, not only centers on the use of neural-modulating agents, but also with agents to remodel the epigenetic chromatin landscape. The application of genome-wide ‘omics’ technologies illuminates that specific epigenetic changes in ASD subjects can facilitate the discovery of new diagnostic biomarkers to enable non-invasive tests (for example, miRNA profiling in infant saliva), holding promise to detect ASD occurrence at a lower age than currently possible. In addition to miRNA, other epigenetic markers, such as histone post-translational modifications and DNA methylation, also hold potential as signature diagnostic indicators. Nonetheless, advancements in the understanding of epigenetic deregulation in ASD is still at the infancy stage. Although further multi-omics studies will certainly uncover more specific epigenetic profiles, a comprehensive genome-wide overview cannot be completed without in-depth functional analyses to reveal pathogenic mechanisms at the molecular level. This is especially the case in clarifying implications of a wide range of genomic changes such as the copy number variation and single-nucleotide polymorphisms in the brain development of ASD individuals. The development of ASD is a multi-factorial issue that encompasses the interplay between multiple gene loci including the non-coding genome. All these are further modulated by the yet largely unknown influence of the environment. Such complex interactions pose significant limitations to the development of therapeutics, which include those discussed in this review. However, some optimistic signs are dawning, with some recent works employing cutting-edge technologies such as patient-derived organoid culture and single-cell RNA sequencing, which have connected ASD development with the haploinsufficiency of epigenetic factors and chromatin remodelers (SUV420H1/KMT5B, ARID1B, and CHD8) [196], while the inhibition of LSD1 demethylase has been observed to reverse ASD phenotypes in mice [197]. Henceforth, combined genome-scale analyses and in-depth functional characterizations—which have fueled our understandings of epigenetic regulatory mechanisms over the past two decades—are expected to enlighten us on the molecular mechanisms of ASD pathogenesis to achieve precision medicine in ASD treatment. Lastly, it should be noted that this review merely provides an overview of the recent developments in epigenetic understandings of ASD pathogenesis and falls short of an in-depth discussion of many of the important aspects of discoveries in the ASD field, which are covered by other excellent publications.

## Data Availability

No new data were created.
